# A qualitative study exploring why adults with intellectual disabilities and obesity want to lose weight and views of their carers

**DOI:** 10.1186/s40608-015-0080-2

**Published:** 2015-12-18

**Authors:** N. Jones, C. A. Melville, L. Harris, L. Bleazard, C. R. Hankey

**Affiliations:** Mental Health and Wellbeing, University of Glasgow, 1st Floor Admin Building, Gartnavel Royal Hospital, 1055 Great Western Road, Glasgow, G12 0XH UK; Human Nutrition Section, University of Glasgow, New Lister Building, Glasgow Royal Infirmary, 10-16 Alexandra Parade, Glasgow, G31 2ER UK

**Keywords:** Intellectual disabilities, Obesity, Weight management, Motivations for weight loss, Carers

## Abstract

**Background:**

Obesity is more prevalent in adults with intellectual disabilities (ID) compared to the general population. Motivations for weight loss may influence engagement with weight management programmes and have only been studied in adults without ID. Aims: To determine reasons given by adults with ID and obesity for seeking weight loss and whether these reasons differ from those of their carers.

**Methods:**

Prior to a multi-component weight management intervention, participants were asked “why do you want to lose weight?” Carers were asked their views and these were compared to the answers given by the adult with ID. Responses were themed. The Fisher’s Exact analysis was used to test for any relationship between reasons for seeking weight loss and participants’ level of ID, age, gender and BMI.

**Results:**

Eighteen men and 32 women; age 41.6 SD 14.6 years; BMI 40.8 SD 7.5 kg/m^2^; Level ID Mild (28 %), Moderate (42 %), Severe (22 %), Profound (8 %). Eleven were unable to respond. Six themes emerged; Health; Fitness / Activity / Mobility; Appearance / Clothes; Emotional / Happiness; For Others; Miscellaneous. The most frequent reason given overall and by women was “appearance.” Carers cited “health” most frequently and “appearance” least, rarely agreeing with participants. “Health” was given as a reason more from older adults and those with milder ID. No statistically significant associations were found between reasons for seeking weight loss and BMI age, gender or level of ID but the differing views of adults with ID and their carers were clear.

**Conclusions:**

Views of adults with obesity and mild or moderate ID can be collected. The opposing views of adults and their carers may affect motivation for weight loss.

## Background

Given the increased prevalence of obesity (and associated co-morbidities) in adults with intellectual disabilities (ID) compared with the general population [1], a number of weight and healthy lifestyle interventions have recently been developed for this group.

Many studies have looked at motivators for intentional weight loss in adults [2–5] and have identified reasons such as health, fitness, appearance and a specific suggestion from a health professional. However, to date, reasons that adults with ID have for seeking weight loss, remain unknown. It has been proposed that specific reasons for wanting to lose weight may act as weight loss predictors [2, 6–10] as well as providing a focus for health consultations [7, 11] and could be important to maximise levels of engagement with weight management programmes.

This study aims to explore the reasons that obese adults with ID give for wanting to lose weight and whether these motivations differ from those given by their carers. This information can contribute to future planning of weight loss interventions in adults with ID, which may maximise engagement.

## Method

### Trial registration

ISRCTN52903778; date applied 30.01.2014; date assigned 12.03.2014

### Design

This study explored the views of adults with ID and obesity and those of their carers via a cross sectional, qualitative design.

Adults with ID and obesity, recruited to a multi-component, weight management intervention study [12] were asked, “why do you want to lose weight?” The question was posed to each participant by one of two researchers (NJ or LB) early in their first contact, prior to delivery of the intervention, in the participant’s home or place of their choice.

After posing the question, time was taken to allow the participant to gather their thoughts and the question re-phrased if it appeared they had not understood. All responses were noted by hand and ranked in order of them being given. In the event of an answer not being given, participants were prompted with questions and discussion until a reason was elicited. For those unable to respond due to communication difficulties or a lack of understanding, “could not answer” was recorded.

The open ended question of “Why do you want to lose weight?” was chosen rather than using closed questions or images. This simple question was repeatable and allowed all views to be secured. During the study design, mock interviews were carried out with adults with a range of ID at a day centre to pilot the question. Individuals provided reasons without the use of images and the images themselves, caused confusion. A closed question, results in many adults with ID acquiescing due to the researcher-participant relationship [13].

Carers present at this session were then asked, “Why do you feel [name of the adult with ID] should lose weight?” Views from carers of those participants unable to respond, were still collected.

All responses were recorded, categorised, themed and the level of agreement between the adult with ID and their carer, determined.

Previous research in adults with ID using qualitative techniques has confirmed the utility of this methodology in this population group [14, 15] and carers [16, 17].

### Ethical considerations

This study complied with the ethical principles of the Declaration of Helsinki and the principles of Good Clinical Practice. Ethical approval was granted for this study within the application for an intervention study on weight management interventions in adults with ID and obesity (A single blind, pilot randomised trial of a weight management intervention for adults with intellectual disabilities and obesity: study protocol) [12] from the Scotland A Research Ethics committee (reference 13/SS/229). Written, informed consent was sought from adults with ID who had the capacity to provide informed consent and, where this was not possible, consent was sought from the nearest relative or welfare guardian.

### Study population

Views of all participants recruited to a multi-component, weight management intervention study [12] were explored in this study. Adults were ≥18 years old (no upper age limit) with a BMI of ≥30 kg/m2 and had an intellectual disability of any level. Further inclusion criteria included being able to walk (with or without a walking aid) for 10 min at a time (based on their own or carer report), not being on a prescribed or restricted diet and not having intentionally lost >3 kg over the previous 3 months. Exclusion criteria included those with Prader Willi syndrome, Cohen syndrome or Bardet-Biedl syndrome, those already taking part in another research study, those taking weight loss medication and anyone who was pregnant at any stage of the study.

### Anthropometric measurements

Participants’ anthropometry and level of ID [13, 18] had been collected as part of the main intervention study [12] by a separate researcher (LH), not involved in the intervention delivery. BMI was measured using the formula BMI = weight/height (kg/m^2^). Weight was measured in light clothing without shoes to the nearest 100 g using SECA877 scales (SE approval class III; SEA Germany). Height was measured to the nearest 1 mm using the SECA Leicester stadiometer (SECA, Germany).

### Level of ID

ID level was available and participants were categorised as having mild, moderate, severe or profound ID as derived from a previous study [18]. This was robust, with a good level of agreement with a validated assessment of ability [13].

### Data anaylsis

The objective of the analysis was to determine reasons given by adults with ID for seeking weight loss and views of their carers. All participants and carers taking part in the study [12] were asked the question and the answers given were recorded at the time, by hand. The qualitative analytic method of thematic analysis was used because it allowed for the identification, analysis and reporting of patterns within the data [19]. Once the data collection was complete and familiarised with, responses were categorised into themes.

### Statistical analysis

Descriptive statistics were performed for participant’s level of ID, age, gender and BMI. Chi-squared (Fisher’s Exact test) analysis was used to examine the relationship between the above variables and participants’ reasons for weight loss.

## Results

Of the 50 participants eligible to participate, 39 (18 male) took part (Table 1) and were included in the analysis. Eleven who could not respond were either of moderate (27 %), severe (46 %) or profound (27 %) disability. Six out of 11 people with severe ID and 1 out of 4 people with profound ID were able to answer the question. Of those with mild / moderate ID, 91.4 % could answer the questions compared to 46.7 % of those with severe / profound ID and this was statistically significant (*p* = 0.001). Of 50 potential carers, 42 answered the question (7 were not present and 1 was unable to provide a reason).

**Table 1:** Participant characteristics

		Male	Female	All	Able to answer question
		*n* = 18	*n* = 32	*n* = 50	*n* = 39
Age		43.6	40.6	41.6	41.5
years (SD)		(15.9)	(14.0)	(14.6)	(14.6)
BMI		42.2	40.5	40.8	41.1
kg/m^2^ (SD)		(8.4)	(7.1)	(7.5)	(7.7)
Level ID	Mild	28	28	28	36
% (n)		(5)	(9)	(14)	(14)
	Moderate	50	38	42	46
		(9)	(12)	(21)	(18)
	Severe	17	25	22	15
		(3)	(8)	(11)	(6)
	Profound	6	9	8	3
		(1)	(3)	(4)	(1)

Participants and carers often provided more than one reason. Six themes emerged 1) health 2) fitness / activity / mobility, 3) appearance / clothes 4) emotional / happiness 5) For others 6) Miscellaneous. The following responses from adults with ID (P) to the researcher’s (R) question “why do you want to lose weight,” demonstrate how participant responses were themed.

- *Health*: P: “For my cholesterol”
- *Fitness / Activity / Mobility:* One participant demonstrated that he could not reach to tie his own shoelaces
- Appearance / clothes: P: “To buy nice clothes for the wedding in November”
- Emotional Happiness: P: “I want to feel happier on my birthday.” R: “Why would losing weight make you feel happier?” P: “because I would have lost weight”
- For Others: P: “My carer told me to (lose weight) for my lungs and my heart”
- The following responses from carers (C) to the researcher’s (R) question “why do you think they should lose weight,” demonstrates how carer responses were themed.
- Health: C (mother): “So that he doesn’t die before I do”
- Fitness / Activity / Mobility: C (paid carer): “To be able to move more and be more active”
- Appearance / Clothes and Emotional Happiness: C (mother): “To feel trendy when she is socialising”
- Emotional happiness: C (paid carer): “To be a happier person”
- For others: C (paid carer): “The doctor told me she needed to lose weight for her blood pressure”
- Miscellaneous: C (brother): “Because he was not this overweight a few years ago”

**Fig. 1:**
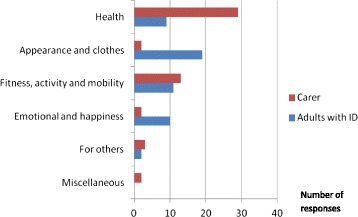
All reasons provided by adults with ID and carers

Figure 1 shows “appearance and clothes” was the most frequent reason given by individuals with ID for weight loss, both overall (accounting for 37 % of all participant responses) and as a primary reason, followed by fitness, activity and mobility; emotional and happiness; health (accounting for 18 % of all participant responses) and lastly, for others. This was in contrast to the views held by carers where 29 carer responses (57 %) were for health and only 2 carer responses (4 %) were for appearance.

Agreement of any reason given by a participant and their carer occurred four times and, in two of those cases, did their first responses match. There was found to be no relationship between the answers given by the participant and their carer (*p* = 0.36).

Men cited fitness / activity / mobility most often overall (35 %), followed by health (25 %) then appearance (25 %). Fitness / activity / mobility was also provided by men most frequently as a primary reason (28 %). Women were more likely to cite appearance / clothes and emotional / happiness (45 % and 23 % respectively) and again, this pattern was similar when looking at women’s first reason (41 % and 13 %). For both men and women, it was uncommon for people to cite “for others” as a reason. There was no significant difference between reasons for weight loss and gender (*p* = 0.20).

Health accounted for 30 % of reasons given by people ≥50 years old compared to 14 % of those given by people between 30 and 49 years old and 6 % of those given by younger adults <30 years. Whereas the importance of health increased with age, the importance of appearance / clothes and emotional happiness decreased. Nearly half (47 %) of all reasons provided by younger adults under 30, was appearance / clothes compared to 30 % of older adults ≥50. Fitness, activity and mobility was most important to those in the middle age group between 30 and 49 years old. There was no significant association between age groups and participants’ reasons for weight loss (*p* = 0.21).

Although there was not a statically significant difference in the reasons for seeking weight loss between different ID level groups (*p* = 0.99), there was a tendency for health to be given as a reason more frequently by those who had mild ID (28 %) compared to those with moderate ID (15 %) and those with severe and profound ID (0 %). Appearance / clothes and fitness / activity / mobility were the only reasons given by those with severe or profound ID.

Appearance and clothes, although a strong motivating factor for weight loss overall, was a particulary common reason in those with a BMI of ≥50 kg/m^2^, accounting for half (3 out of 6) of all reasons in this BMI group. Only those in the lower BMI range of 30–39 kg/m^2^, cited “for others” as a reason for losing weight. By the higher BMIs (≥40 kg/m^2^), all individuals that were able to answer the question, had reasons for wanting to lose weight for themselves, rather than for others. However, there was no significant association between classification of obesity and participants reasons for wanting to lose weight (*p* = 0.68)

## Discussion

This is the first study in individuals with ID and obesity exploring reasons for weight loss. It has illustrated the feasibility of qualitative work in adults with ID, using established methods [10, 11, 14, 17, 20]. Of those who responded, 82 % had a mild or moderate ID. It was not possible for all of those with more severe ID to make their views heard. The opposing views held by adults with ID and their carers were clear.

### Appearance is a greater motivator for weight loss in adults with obesity and ID than health and there is a lack of agreement between adults with ID and their carers

This study agrees with others [16, 17] that health is a priority for carers. Carers’ perspectives of a weight loss intervention for adults with ID [17], revealed a theme of “carers perceptions of participants health”, including “reasons to lose weight.” Carers reported health, mobility and psychosocial factors as reasons why their client should lose weight. These mirrored a previous study [16] where carers were found to recognise the health benefits of healthier lifestyles for adults with ID, more readily than self image or quality of life benefits.

Although it has been suggested [11, 17] that, ascertaining views of carers of adults with ID is a reasonable way to reflect experiences of this population group, this study has demonstrated a lack of agreement between adults with ID and their carers. The difference in the wording of the question to the participant (asking why they would *want* to lose weight), compared to that asked of the carer (why they felt the participant *should* lose weight) was necessary as the carer would have heard the participant’s answer before they responded. This wording difference is unlikely to have contributed to the lack of agreement.

Carers have a pivotal relationship with the person they support and impact on compliance to many activities, including health interventions such as weight management [17]. Spanos et al (2012) found that discussing health benefits of weight loss, although a strong motivator for carers, was unsuccessful at motivating the participant. It was suggested that motivational goals are pertinent to each individual and may be more related to day to day priorities rather than health *per se* [17]. This study extends this knowledge, demonstrating that appearance and clothes are important motivating factors for adults with ID, particularly for women and adults with BMIs of ≥50, possibly reflecting the difficulties in finding clothes in larger sizes. “Appearance / clothes” and “fitness / activity / mobility” were the only reasons given by those with severe or profound ID, perhaps reflecting the immediate discomfort that these effects of weight give them, compared to the relatively abstract and more complicated notion of health.

Although health was not a strong motivating factor for adults with ID overall in this study, the frequency of citing “health”, increased with age, perhaps reflecting the greater health problems, medical consultations and medications associated with increasing age. Health was also cited more by those with milder ID, possibly due to them having a greater understanding of the link between weight and health and of their carer’s views prior to the question being asked. The men studied by Hankey et al (2001), were motivated primarily by health [3] and it is possible that the difference in motivation between adults with ID and those without is due to the degree of understanding of the link between weight and health.

These findings suggest carer’s awareness of their client’s motivating factors is poor and that views of the adult with ID differ from their own. Using “appearance” and “clothes” as topics of conversation surrounding weight loss and healthy lifestyles, could help initiate motivation to make lifestyle changes.

### Motivating factors for weight loss differ between adults with obesity and ID, and those without ID

Previous studies exploring reasons that people without ID choose to lose weight have found “health” to be the most frequently reported [6, 8, 21–25]. Medical triggers, a recent health problem and physical symptoms resulting from excess body weight were found to stimulate behaviour change [8, 23], achieve greater initial weight loss and result in reduced weight re-gain [2, 6]. In successful weight losers, defined as having lost 10 % of their body weight and having kept it off for 3 months, a health anxiety prompted by a new health problem or diagnosis, was found to be their primary reason for wanting to lose weight, followed by the advice of a health professional and lastly appearance [10]. The main difference between reasons cited in the lower BMI range in adults with ID (30–39.9 kg/m^2^) in this study and men without ID of the same BMI [3] was that health followed by fitness was the most frequent response in men without ID. In this study, there were 8 men with a BMI of 30–39.9 kg/m^2^ and between them provided 8 responses, equally split between fitness / activity / mobility, appearance / clothes, emotional / happiness and health.

Men [6, 8, 25] and particularly older men between 40 and 55 years old [3] seem from the literature to be more likely than women to cite health or medical triggers as a reason to intentionally lose weight. Two studies [21, 24] found no gender differences in reasons to lose weight, though the latter study examined reasons in a specific group of those diagnosed with binge eating disorder.

Appearance as a reason for weight loss was mentioned as a primary reason in women [8, 25], in younger men [3] and younger adults generally [2] and a secondary reasons in others [3, 8, 10, 22–24]. Overall, it appears that appearance is a greater motivator for younger people to lose weight [2, 3, 26] and it is not until the age of thirty five [2] or forty years old [3] that people want to lose weight for health reasons.

Emotional reasons such as “mood” or “psychological reasons” were mentioned infrequently in the papers identified [22, 23].

It is debateable whether having health or appearance as a motivating factor for weight loss is more beneficial in terms of long term weight loss maintenance. Roberts et al (1999) suggest that, over time, a health anxiety which may have initiated the behaviour change for weight loss may be replaced in part by the positive rewards of perceived improved appearance and fitness, enabling the person to sustain any behaviour change and thus sustain weight loss long term [10]. However, Lawrence et al (2001) warns against using short term weight loss triggers such as a forthcoming social event as the behaviour changes are harder to maintain once the event has passed [8].

It is uncommon for men without ID to cite “for others” as a reason for wanting to lose weight [3] and this was also the case for the adults with ID in this study. In this study, only those in the lower BMI range of 30–39 kg/m^2^ cited “for others” as a reason for seeking weight loss and this lack of internal motivation in some could possibly reflect a lack of awareness, among the less obese individuals with ID, of the need to lose weight.

### It is necessary and possible to ascertain views of adults with mild to moderate ID

With the emphasis on adults with ID having an active role in their lifestyle choices, goal setting and actions [17], it is becoming increasingly clear that we need a greater understanding of their views in order to inform future service provision. The literature suggests that obtaining views from adults with ID, although important in order to understand their experiences, views and aspirations [11, 15, 20], can be problematic [11, 14]. However, Beail and Williams (2014), conclude that, “If we want to hear the voices of people who have ID, then we need appropriate ways to do this” [20].

It has been said that, “The emphasis on research should be on overcoming the barriers that impede the involvement of inarticulate subjects instead of highlighting the difficulties they present,” [14]. Suggestions to facilitate communication and obtaining of reliable responses include using images, taking adequate time [15], considering the pros and cons of open versus closed questions, taking note of non-narrative communication and exploring different modes of questioning [14]. It may be the case that, for this patient group, the researcher needs to use different methods of questioning and conversation for different individuals and different methods compared to those used in qualitative research in adults without ID. “Loaning words” for example would be seen as putting words into a person’s mouth in research with adults without ID but may be necessary as part of options and general conversation for adults in this patient group [14].

### Limitations

Although all individuals participating in the weight management intervention study [12] were asked their views, those with severe and profound ID were less able to provide an answer.

There was no significant association between reasons for weight loss and gender, age, ID and BMI and this could be partly due to the small sample size. The fact that 11 people could not express a reason, meant that a potential sample of 50 individuals with ID was reduced to 39.

There was no deeper exploration as to why a particular response was given and this could have led to a more thorough understanding of the reason for seeking weight loss. Using resources such as “talking mats” [27] and taking more time over the question, could lead to gaining further insight into the data.

### Future work

Whether reasons for weight loss impact on weight outcomes in obese adults with ID is of interest given previous studies that have found such relationships in obese adults without ID [2, 6–10], as is the effect of participant–carer discrepancies. It would be beneficial to determine techniques to understand views of individuals with severe and profound ID.

## Conclusion

Obese adults with mild and moderate ID who were recruited to a weight management study, were able to express their views on their reasons for wanting to lose weight. This was increasingly harder for those with more severe ID. Findings show that the individuals with ID were particularly interested in their appearance, a view rarely shared by carers. Reasons for weight loss may have a role in maximising engagement opportunities with weight management interventions for those with ID and obesity.

## Abbreviations

%: percent
BMI: Body mass index
ID: Intellectual disabilities
kg: kilogram
SD: Standard Deviation
